# Mosaic *PKHD1* in Polycystic Kidneys Caused Aberrant Protein Expression in the Mitochondria and Lysosomes

**DOI:** 10.3389/fmed.2021.743150

**Published:** 2021-12-16

**Authors:** Chengxian Xu, Chenxi Yang, Qing Ye, Jie Xu, Lingxiao Tong, Yuchen Zhang, Huijun Shen, Zhihong Lu, Jingjing Wang, Enyin Lai, Jianhua Mao, Pingping Jiang

**Affiliations:** ^1^Department of Nephrology, The Children's Hospital, Zhejiang University School of Medicine and National Clinical Research Center for Child Health, Hangzhou, China; ^2^Institute of Genetics and Department of Human Genetics, Zhejiang University School of Medicine, Hangzhou, China; ^3^Department of Physiology, School of Basic Medical Sciences, and Kidney Disease Center of the First Affiliated Hospital, Zhejiang University School of Medicine, Hangzhou, China; ^4^Zhejiang Provincial Key Laboratory of Genetic and Developmental Disorders, Hangzhou, China

**Keywords:** ARPKD, exosome, lysosome, mosaicism, PKHD1, mitochondria

## Abstract

Autosomal recessive polycystic kidney disease (ARPKD) is a severe renal cystic disease caused mainly by the polycystic kidney and hepatic disease 1 (*PKHD1)*. However, the genetic cause, pathologic features, and mechanism of action of ARPKD are not well known. Here, we identified a family with ARPKD. Two siblings harbored biallelic variants in *PKHD1* (c.7205G>A, c.7973T>A). We determined that the “*de novo*” variant, c.7205G>A, arose from the mosaicism of the father and had a 7.4% level. Pathologic characterization, using biopsy analysis, was evidenced with predominant cystic dilation in proximal tubules, slight ectasia of collecting ducts, defective ciliogenesis, and impaired cell-cell junctions in renal tubules and collecting ducts. Exosome proteomics in the urine from patients with ARPKD were markedly different from those of controls, with the most significant alterations occurring in mitochondrial and lysosomal proteins. Expression of the proteins of OXPHOS was downregulated sharply, in parallel with upregulated expression of the proteins involved in glycolysis in patients with ARPKD. Several lysosomal proteins associated with renal lesions were more abundant in the exosome of the patient than in controls. Moreover, the lysosomal enzyme sulfamidase, which is produced by the *SGSH* gene, was abrupt uniquely in the exosome of the patient. Consistently, swollen mitochondria and abundant lysosomes were visualized in the mutant tubular epithelial cells of patients with mutant *PKHD1*. Collectively, these findings provide new insights on the pathophysiology of the polycystic kidney due to PKHD1 deficiency. *PKHD1* mosaicism should be considered in genetic testing of ARPKD patients.

## Introduction

Autosomal recessive polycystic kidney disease (ARPKD) is a rare and severe renal cystic disease caused by homozygous or compound heterozygous variants of polycystic kidney and hepatic disease 1 (*PKHD1*) or DAZ interacting zinc finger protein 1 like (*DZIP1L*) (1, 2). ARPKD is characterized by fusiform cysts in the collecting ducts of the kidney. The major defect of PKHD1 is postulated to result in dedifferentiation of cells, excessive secretion of fluid, and proliferation of tubular epithelia causing renal cysts (3).

However, the use of a large membrane spanning protein, fibrocystin/polyductin (FPC), is challenging for exploring the pathogenesis of *PKHD1* variants in ARPKD. Urinary extracellular vesicles (including exosomes) are considered to be “reservoirs” of altered proteins during PKD pathogenesis, in which many urinary proteins have been identified to be associated with kidney diseases or renal lesions, such as aquaporin 2 (AQP2), uromodulin, and beta-galactosidase (BGAL) (4).

In this study, we present a rare case of ARPKD due to compound heterozygous *PKHD1* variants, of which one “*de novo*” variant was originally from the mosaicism of the father. Proteomic analysis and immunohistochemical (IHC) analyses of exosomes revealed significant alternative profiling of mitochondrial and lysosomal proteins in patients with ARPKD and an abrupt increase in expression of lysosomal N-sulfoglucosamine sulfohydrolase (SGSH) in both urinary exosomes and renal biopsies.

## Materials and Methods

### Ethical Approval of the Study Protocol

This study was conducted in accordance with the World Medical Association Declaration of Helsinki (version 2008). Written informed consent, blood samples, and clinical evaluations were obtained from patients and/or their parents under a protocol approved by the Research Ethics Committees of the Children's Hospital, Zhejiang University School of Medicine (Hangzhou, China).

### Participants

A Han Chinese family with ARPKD (Supplementary Table S1) was identified at the Children's Hospital, Zhejiang University School of Medicine.

### Whole-Exome Sequencing and Validation of Mosaic PKHD1

Genomic DNA was isolated from peripheral blood with a DNA Blood Mini kit (catalog number, 51104; Qiagen, Stanford, VA, USA). A library of whole exomes was constructed using the Nimble Gen Seq EZ Exome Enrichment kit and its capture probes (Roche, Basel, Switzerland), followed by 150-bp paired-end sequencing with NovaSeq 6000 (Illumina, San Diego, CA, USA). Clean data were aligned to the GRCh37/hg19 using the Burrows–Wheeler Aligner-Maximal Exact Match (BWA-MEM) algorithm (5). Variants were called by the HaplotypeCaller (https://gatk.broadinstitute.org/) (6) and annotated with databases such as Exome Aggregation Consortium (ExAC) ect. and predicted by online analysis programs. Mosaic samples were detected by a unique molecular identifier (UMI) and deep sequencing according to the ABI BigDye™ Terminator Cycle Sequencing kit (4376484; Applied Biosystems, Carlsbad, CA, USA) with an ABI3130XL analyzer (Thermo Fisher Scientific, Waltham, MA, USA). The reliability of variants was assessed by the ratio of mutated reads and mutated depth between the subjects and the controls. Primers for Sanger sequencing are summarized in Supplementary Table S2. The *PKHD1* variants identified in family members were present in Supplementary Table S3.

### Histology and Immunohistochemistry/Immunofluorescence

Renal-biopsy tissues from two unrelated individuals exhibiting a small glomerular lesion without tubular modification were used as controls. For staining, sections of thickness 5 μm were sliced and stained with hematoxylin and eosin (H&E). For IHC analyses, paraffin-embedded sections were deparaffinized and blocked in 5% fetal bovine serum (FBS) for 30 min. This action was followed by incubation with primary antibodies overnight at 4°C, the secondary biotinylated antibody for 2 h at room temperature, and, finally, peroxidase-conjugated streptavidin (1:10,000 dilutions; ab7403; Abcam, Cambridge, UK) for 10 min. For immunofluorescence staining, sections were blocked in 5% bovine serum albumin–phosphate-buffered saline for 30 min, and incubated with primary antibodies and then secondary antibodies. The antibodies we used are listed in Supplementary Table S4. Images were recorded by a microscope (DM4000; Leica, Wetzlar, Germany).

### Assessment of Exosome Proteomics

Two patients (II-1 and II-2) carrying *PKHD1* variants, and two age-matched unrelated controls, were recruited for analysis. Exosomes were prepared as described elsewhere (7). Liquid chromatography-tandem mass spectrometry (LC-MS/MS) was done on a Q Exactive™ Orbitrap mass spectrometer (Thermo Fisher Scientific, Shanghai, China) coupled to an Easy nLC system (Thermo Fisher Scientific). MaxQuant (www.maxquant.org/) was used for protein identification and label-free quantitative proteomics analysis (8). Gene ontology (GO) terms were mapped by BLAST+ (National Center for Biotechnology Information, Bethesda, MD, USA). Blast2GO (9), Kyoto Encyclopedia of Genes and Genomes (KEGG) database (www.genome.jp/kegg/) (10), and Human MitoCarta3.0 datasets (https://www.broadinstitute.org/files/shared/metabolism/mitocarta/human. mitocarta3.0.html) were also used for protein analysis. Differences were considered significant if fold change (FC) ≥2 and *P*-value <0.05. The FC was assigned to “−1” in the heatmap when protein was undetectable.

### Transmission Electron Microscopy

Biopsy specimens were fixed in 2.5% glutaraldehyde overnight at 4°C, rinsed with phosphate-buffered saline, and post-fixed in a 1% osmic acid for 2 h. Then, samples were dehydrated in a graded series of ethanol solutions, permeated in acetone and Spurr resin, and embedded in epoxy resin. Ultrathin (70 nm) sections were obtained using a microtome (EM UC7; Leica (Leica microsystem, Shanghai, China)) and then stained with uranyl acetate and lead citrate. Images of mitochondrial ultrastructure were captured by a transmission electron microscope (H-7650; Hitachi, Tokyo, Japan) with ImageJ (US National Institutes of Health, Bethesda, MD, USA) (11).

### Statistical Analyses

Data were analyzed using Prism 8.00 (GraphPad, San Diego, CA, USA) and are presented as the mean ± SD. The unpaired, two-tailed Student's *t*-test was carried out between two groups. *P* < 0.05 was considered significant.

## Results

### Case Presentation and Identification of a Mosaic PKHD1 Variant

**Figure 1:**
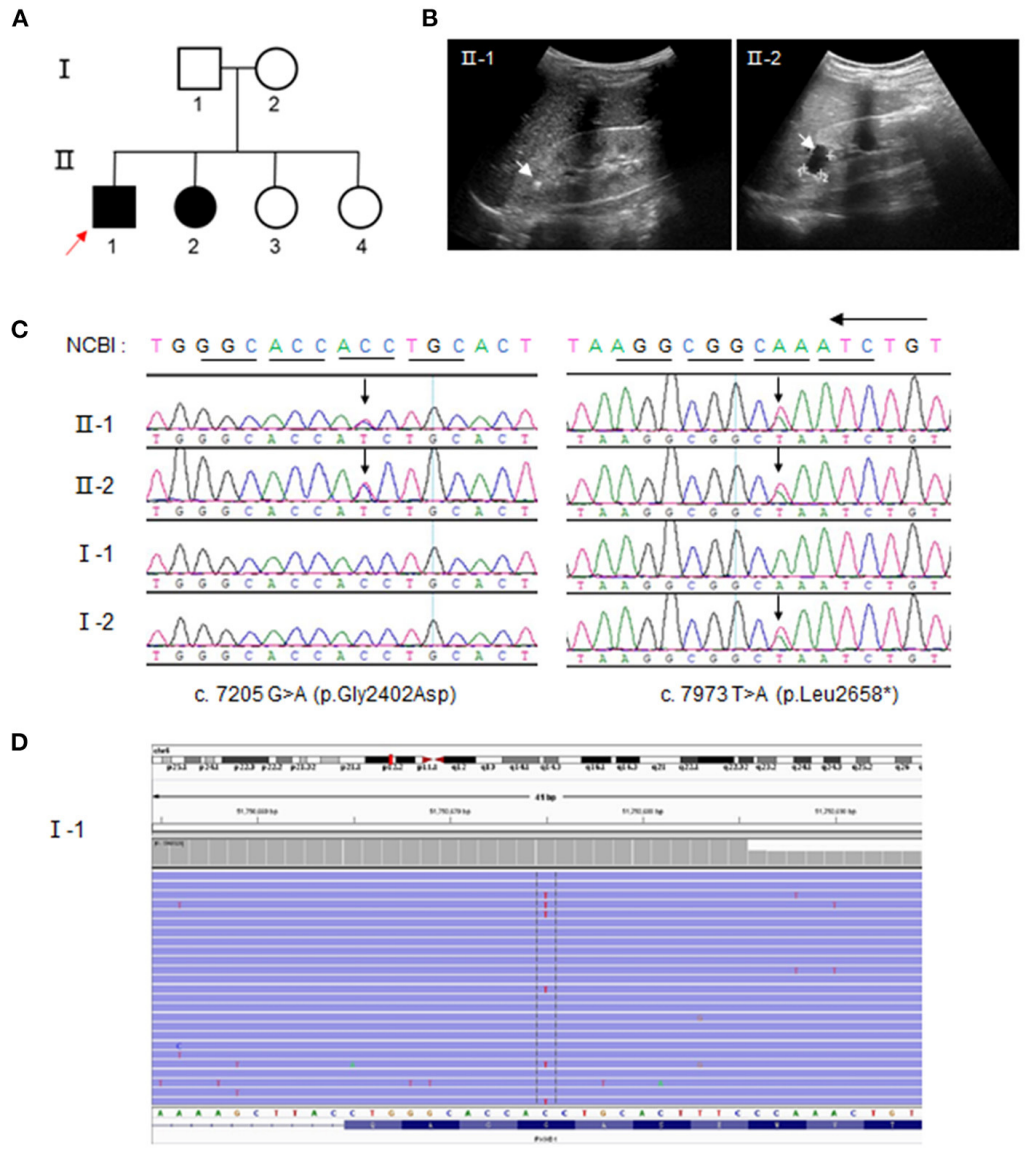
Compound heterozygous polycystic kidney and hepatic disease 1 (*PKHD1*) variants from parental mosaicism in a family with autosomal recessive polycystic kidney disease (ARPKD). **(A)** ARPKD pedigree. Squares = men; circles = women; Solid line = ARPKD patients; red arrow = proband. **(B)** Ultrasound images showing hyperechoic crystals and cysts in family members II-1 and II-2, respectively. **(C)** Identification of variants, c.7205 G>A and c.7973 T>A, by Sanger sequencing. The arrow indicates the position of the mutant base. **(D)** Unique Molecular identifier (UMI) and targeted-capture next-generation sequencing analysis (20,000×) for the low level of this c.7205 G>A (p. Gly2402Asp) variant, ~7.404%, in the PBL of the father (I-1). The red base in the center of the reads denotes the “C” mutation to “T”.

One kindred with ARPKD was referred to our institution for further treatment (Figure 1A). The proband (II-1) was a 6-year-old boy who was referred to our nephrology clinic for consultation due to recurrent periumbilical pain, abnormal urinalysis, and hypertension (Supplementary Table S1). He presented with mild proteinuria (10–25 mg/kg·d), microalbuminuria, and microhematuria ranging from 32 to 91 RBC/HP (normal interval, 0–3 RBC/HP). His serum creatinine was 44 μmol/L, and eGFR 105 ml/min/1.73 m^2^. The 24-h ambulatory blood pressure was monitored with a mild elevation (145–109/97–66 mmHg). The images of B ultrasound revealed bilateral hyperechoic renal cortex (Figure 1B). No significant cyst was found by B ultrasound nor by Magnetic resonance image at present time.

The elder sister (II-2) was 11-year-old and diagnosed to be ARPKD based on the urinalysis and gene testing. She presented with microhematuria (12 RBC/HP). No hypertension and proteinuria were found at present time. However, multiple renal cysts and bilateral hyperechoic renal cortex were manifested in the image of B ultrasound (Figure 1B), whereas no abnormal findings were detected in the liver or bile duct by B ultrasound. Other family members were healthy, without the involvement of the liver or kidney.

Two variants, a missense one c.7205G>A (p. Gly2402Asp), and a truncated one c.7973T>A (p. Leu2658^*^), were identified by whole-exome sequencing in DNA from peripheral blood lymphocytes (PBL) from the affected proband (II-1) and confirmed by Sanger sequencing (Figure 1C; Supplementary Table S3). Other family members were screened to determine the origin of the variants. The same compound heterozygous variants were detected in the affected sister (II-2), whereas the mother (I-2) and her two elder sisters (II-3 and II-4) carried only one variant: c.7973T>A. Strikingly, no variant was detectable in the sample of peripheral blood lymphocytes from the father according to Sanger sequencing at first. To explore whether the variant c.7205G>A was “*de novo*,” UMI and the targeted next-generation sequencing were carried out. We determined a 7.4% level of c.7205G>A in the father, which indicated a germinal mosaic origin (Figure 1D).

### Defects of Renal Tubules, Primary Cilia, and Cell-Cell Junctions in Renal Biopsies

**Figure 2:**
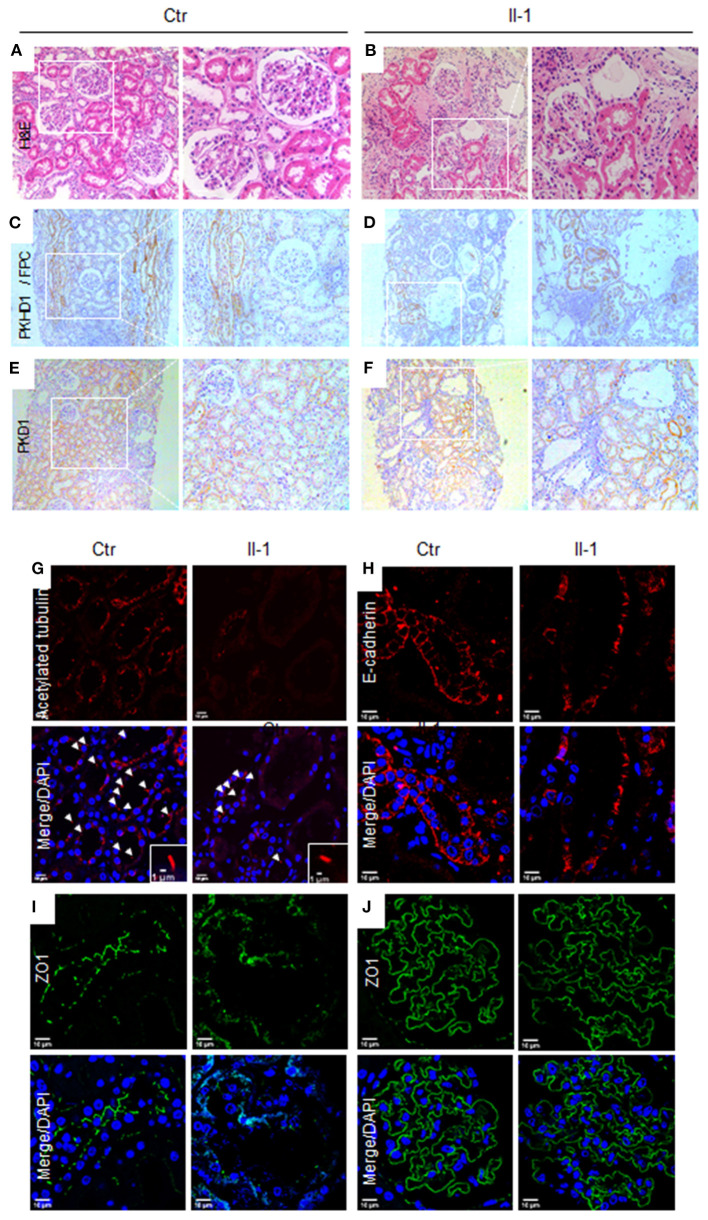
Pathological features in biopsies for patients with the PKHD1 mutant. **(A,B)** H&E staining, illustrating patchy ectasia in the proximal tubules and flattening of the tubular epithelial cells in dilated tubules from proband (II-1) biopsy specimens, as compared to that of controls. **(C,D)** Immunohistochemical staining of PKHD1/FPC in the collecting ducts of control and patient biopsies. **(E,F)** Immunohistochemical images of PKD1. A detectable difference was not revealed between tissue sections from the control or patient. Magnification: **(A,C)**, and **(E)**, ×100; **(B,D)**, and **(F)**, ×200. **(G)** Immunofluorescence detection of cilia by acetylated α-tubulin (red), counterstained with DAPI. **(H, I)** Analysis of cell-cell junctions by immunofluorescence staining against E-cadherin **(H)**, red, and ZO-1 **(I)**, green, in renal tubular epithelial cells. **(J)** Glomerulus images by staining with ZO-1. No significant difference was detected between biopsies in patients and controls. Scale bar for immunofluorescence, 10 μm. Ctr, control; Proband, II-1.

To investigate the pathological influence of a *PKHD1* mutant on renal structure, we conducted H&E staining and IHC staining for FPC using biopsies. H&E staining revealed significantly dilated proximal renal tubules, surrounded by prominent inflammatory infiltrates and fibrosis in tissue sections (Figures 2A,B). Renal tubular epithelia cells trended to be flat in dilated tubules, and demarcation of the cortex and medulla was less clear in *PKHD1*-mutant specimens. FPC was expressed specifically in collecting ducts (Figure 2C), as reported previously (12). Slight ectasia and misaligned collecting ducts were visualized in *PKHD1*-mutant tissue sections with low expression of FPC (Figure 2D). These data confirmed that the missense mutation, c.7205G>A (p. Gly2402Asp), did not abrogate PKHD1 expression completely (3). No difference in PKD1 expression was found in tissue sections (Figures 2E,F).

Fibrocystin/polyductin has been postulated to maintain cilia function to govern renal-tubule morphology and prevent cyst formation (13). Compared with the control, the biopsy from *PKDH1* mutants showed few primary cilia in the tubular epithelia (Figure 2G). The “adherence junction” pathway is destroyed *in vitro* in null-*PKHD1* cells (14), so we explored the features of cell-cell junctions using the junctional markers E-cadherin and zonula occludens (ZO)-1. Discontinuous junctions were present in the renal tubules and collecting ducts of the *PKHD1* mutant relative to that in the control (Figures 2H,I). Conversely, there was no significant difference in the ZO-1 expression in glomeruli (Figure 2J).

### Alternative Protein Profiling of Mitochondria and Lysosomes in Exosomes

**Figure 3:**
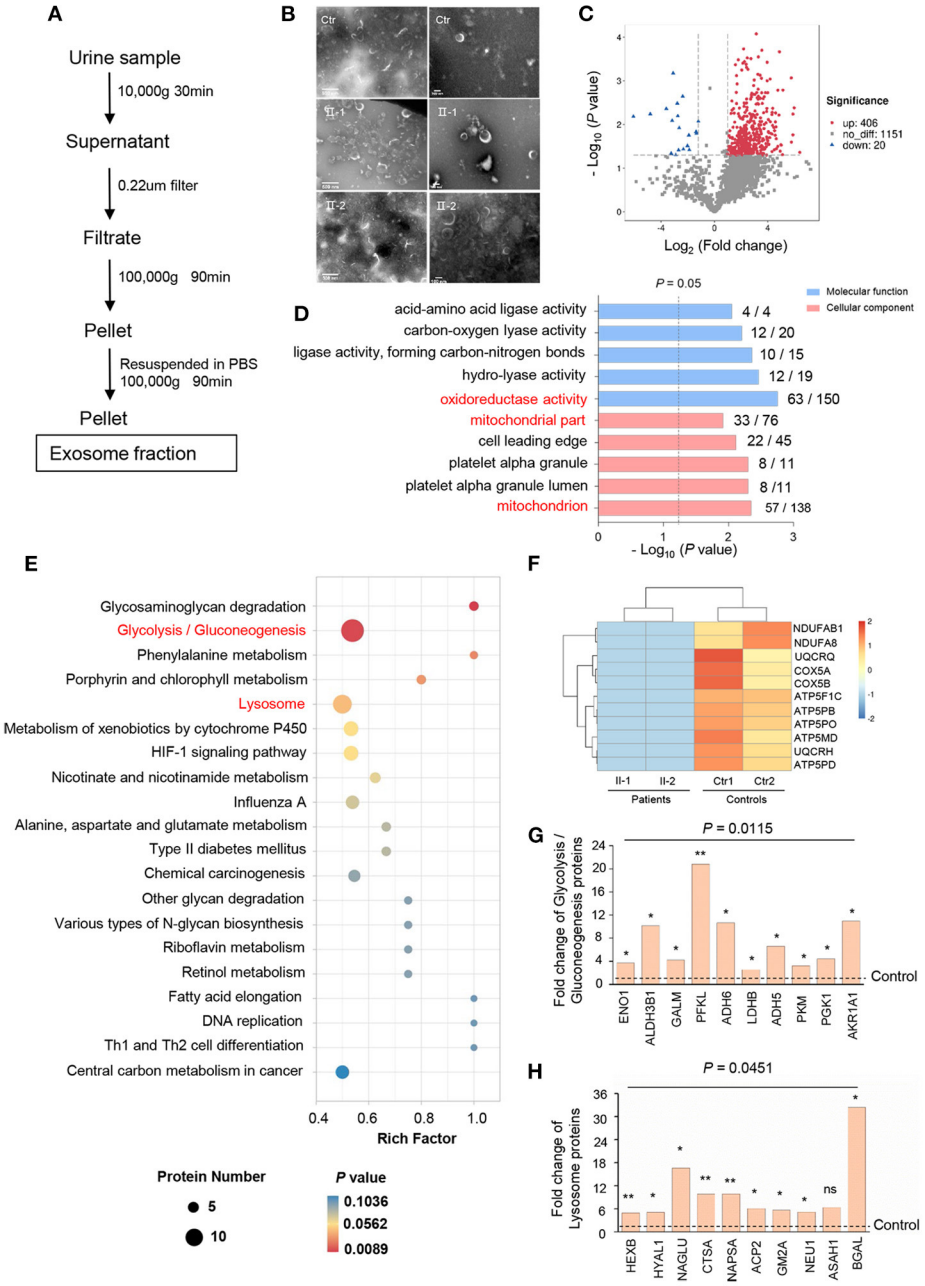
Alterative protein profiling in urinary exosomes. **(A)** Isolation of urinary exosomes. **(B)** TEM images of exosomes from controls (Ctr) and patients (II-1 and II-2) urine. Exosomes have a cup-shaped or round “dimple” appearance. Scale bar: left, 500 nm; right, 100 nm. **(C)** Volcano plots of proteins with significant expression in patients compared with that in controls. Red points, FC ≥2; blue points, FC ≤0.5; gray, not significant. **(D)** GO annotation in molecular function and cellular component (ranked by –log_10_ [*P*-value]) using Blast2Go. The number of significantly expressed proteins and all proteins identified in each GO term were noted. **(E)** Analysis of urinary-exosome proteomics using the KEGG database, showing significant alteration in expression of the proteins of glycolysis/gluconeogenesis, and lysosome pathways between patients and controls. **(F)** Heatmap for downregulation of OXPHOS proteins in patients relative to that in controls. Eleven proteins of OXPHOS were undetectable in *PKHD1-*mutant samples and assigned as “−1”. **(G, H)** Fold-change of individual proteins in the pathways of glycolysis/gluconeogenesis **(G)** and lysosomes **(H)**. ^*^P < 0.05; ^**^P < 0.01.

Urinary exosomes from patients (II-1 and II-2) and controls were collected based on typical ultracentrifugation protocols (Figure 3A) and confirmed to be cup-shaped or to have a “dimple” appearance by TEM (Figure 3B). LC-MS/MS revealed 1,999 proteins to have alternative expression between patients and controls: 1,577 proteins in both groups, 139 unique in patients, and 283 unique in controls, of which one-sixth of proteins were resident in mitochondria. Volcano plots analysis revealed 406 proteins to have significantly upregulated expression and 20 proteins to have significantly downregulated expression (FC ≥ 2, *P* < 0.05) (Figure 3C). In terms of GO annotation, “oxidoreductase activity” in molecular function (*P* = 0.0017) and “mitochondria” in cellular component (*P* = 0.004463) were enriched (Figure 3D), data which reflect that mitochondria are critical for the maintenance of normal renal function (15).

According to the KEGG database, various signaling pathways were altered notably by PKHD1 deficiency: “glycosaminoglycan degradation,” “glycolysis/gluconeogenesis,” and “lysosome” (Figure 3E); these were associated with “energy metabolism,” “amino acid metabolism,” and “autophagy,” respectively. Interestingly, expression of oxidative phosphorylation (OXPHOS) proteins of the respiratory chain in the patients was downregulated markedly compared with that in controls (Figure 3F). Conversely, expression of key enzymes involved glycolysis, such as enolase 1 (ENO1), aldehyde dehydrogenase 3 family member B1 (ALDH3B1), and phosphofructokinase-liver type (PFKL), were upregulated significantly, ranging from 4- to 21-fold (Figure 3G). Additionally, lysosomal proteins were more abundant in the exosome of the patient than in control, of which expression of hexosaminidase subunit beta (HEXB), N-acetyl-alpha-glucosaminidase (NAGLU), and galactosidase beta 1 (BGAL) was increased markedly in patients (Figure 3H). Moreover, the lysosomal enzyme sulfamidase, which is produced by the *SGSH* gene, was abrupt uniquely in patients.

### Increased Expression of SGSH and Swollen Mitochondria in Renal Biopsies

**Figure 4:**
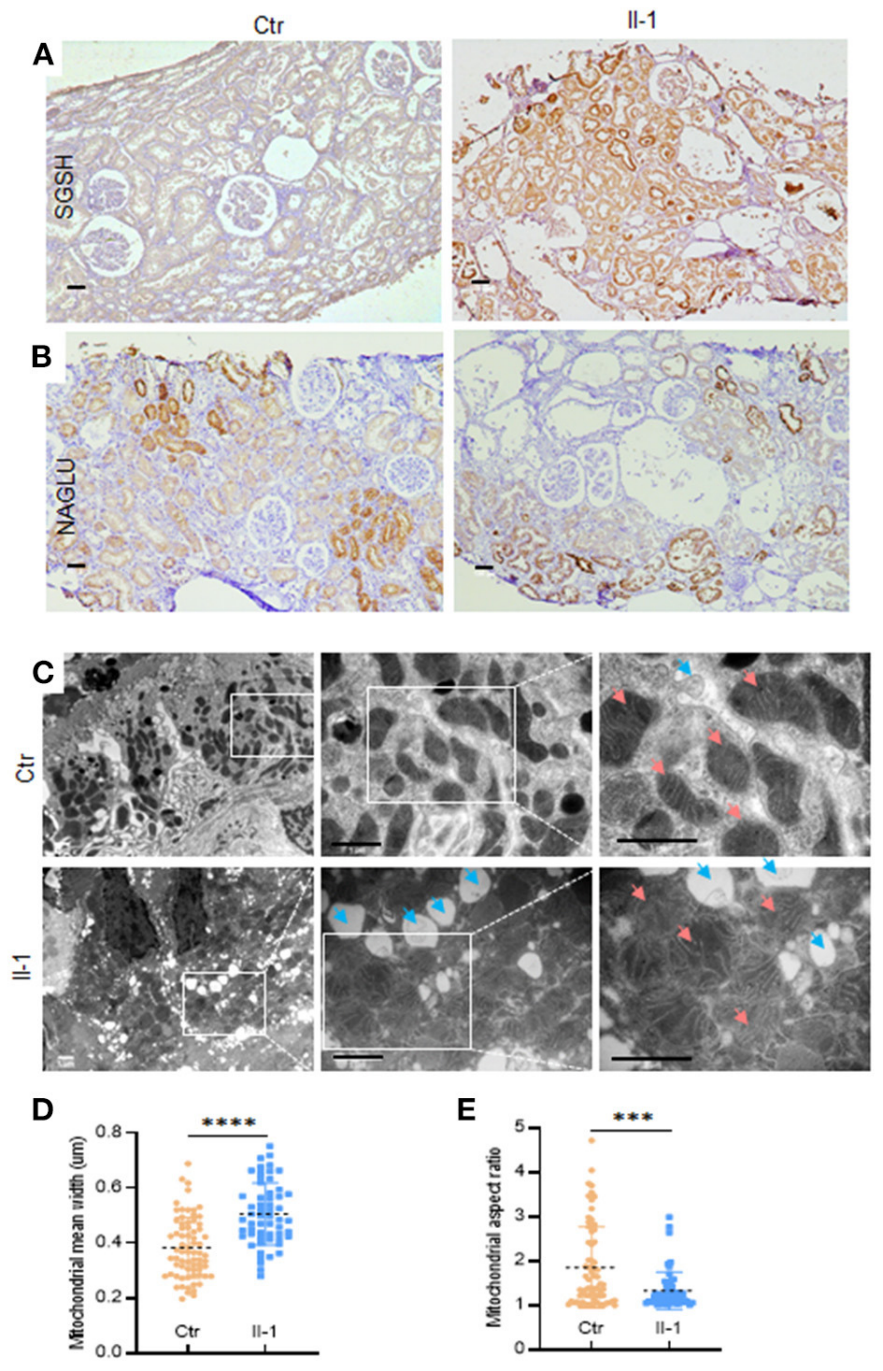
The expression of SGSH in biopsies and TEM of mitochondria and lysosomes in tubular epithelial cells. **(A)** Immunohistochemical staining of SGSH in control (Ctr) and proband (II-1), scale bar = 100 μm. **(B)** Immunohistochemical staining of NAGLU in control (Ctr) and proband (II-1), scale bar = 100 μm. **(C)** TEM images of proximal tubular epithelial cells from the kidney biopsies of controls and patients with ARPKD. Blue arrows = lysosomes; pink arrows = mitochondria. Scale bar = 1 μm. **(D)** Quantification of mitochondrial width at ×30,000 magnification. **(E)** Quantification of mitochondrial aspect ratio. Calculations were based on three random high-power fields (×30,000). Each dot denotes the value of a single mitochondrion. Data are the mean ± SD. ^***^P <0.001 and ^****^P <0.0001.

We wished to ascertain if the excessive secretion of SGSH was also exhibited in *PKHD1*-mutant tissue. Renal-biopsy sections were used for IHC staining against SGSH in parallel with NAGLU antibody (a sensitive indicator of renal lesions) (4). Increased expression of SGSH was detected in the mutant biopsy compared with that in the control (Figure 4A). The expression profile of NAGLU was similar in patients and controls (Figure 4B). Considering the significant differences in protein profiling of lysosomes and mitochondria, we carried out TEM to explore if lysosomes or/and mitochondria in tubular epithelial cells had pathologic changes if PKHD was deficient. Many circular vacuoles were noted among swollen mitochondria in the cells of patients (Figure 4C), which implied that abundant lysosomes had emerged to remove defective mitochondria (15). Besides the disarrangement and loss of cristae (Figure 4C), the mutant mitochondria exhibited an increased mean width (0.5061 vs. 0.3837 μm, *P* < 0.0001) (Figure 4D) and decreased aspect ratio (1.332 vs. 1.855, *P* = 0.0001 (Figure 4E) compared with that in controls, as reported in dysfunctional mitochondria previously (16).

## Discussion

We identified a mosaic *PKHD1* variant associated with ARPKD and found significant alterations of protein profiling in lysosomes and mitochondria by exosomes analysis of urine from a patient with ARPKD. Mosaicism is an unrecognized source of previously considered *de novo* variants in several types of inherited diseases. It has been reported that parental mosaicism accounts for ~10% of *de novo SCN1A* (sodium voltage-gated channel alpha subunit 1) variants in children with Dravet syndrome (17). In the present study, the *de novo* variant, c.7205G>A (p.Gly2402Asp), was found to be of mosaic origin from the father of the patient, implying that mosaicism may be underestimated in ARPKD if the variants' percentage is below the analytical sensitivity (10–20%) of Sanger sequencing (18). The compound heterozygous variants were pathogenic, as evidenced by cystic dilation of proximal tubules and slight ectasia of collecting ducts. Similar ectasia of proximal tubules has been described in heterozygous mice for the *PKHD1*^*C*642*^ variant (19). Conversely, other patients with ARPKD have been shown to have cysts arising predominantly from collecting ducts (3). In the present study, renal lesions were also illustrated by defective ciliogenesis as well as impaired cell-cell junctions in tissue sections from patients, which indicated the complex development of renal cysts.

Exosome proteomics in the urine from patients with ARPKD were markedly different from those of controls, with the most significant alterations occurring in mitochondrial and lysosomal proteins. Expression of the proteins of OXPHOS was downregulated sharply, in parallel with upregulated expression of the proteins involved in glycolysis in patients with ARPKD. These data were in accordance with the abnormal mitochondrial structure observed by TEM. Similar changes in mitochondrial morphology and energy metabolism have been detected in *PKD1*-mutant mice (20), and in HEK-293 cells carrying truncating variants of *PKHD1* or *PKD2* (16). Given the key role of mitochondrial dysfunction in kidney symptoms, our data may explain the predominant dilation of proximal tubules carrying *PKHD1* variants because they are dependent on the efficiency of OXPHOS to generate adenosine triphosphate (ATP) and lack the capacity to produce ATP *via* anaerobic glycolysis (15, 21). Recently, *Pkd1*-deficient murine cells were found to have ~18-fold less effective aerobic glycolysis even in the presence of oxygen (21). Therefore, proximal tubules seemed to be more vulnerable to mitochondrial insults than the collecting ducts in the patients with ARPKD in the present study. Strikingly, the expression of 10 proteins in the lysosome pathway was upregulated by more than five-fold in the exosomes of patients, which confirmed that lysosomes were involved in ARPKD pathogenesis because removing damaged mitochondria *via* mitophagy is extremely important. In the present study, tubular epithelial cells had more lysosomes in biopsies from patients with the mutant *PKHD1*. In addition, the enzyme sulfamidase for lysosomes was abundant in the biopsies of patients with mutant *PKHD1* and was detected specifically in the exosomes in urine, which implied that SGSH could be a urinary biomarker for ARPKD. In contrast, PKHD1 products were not detected in the urinary exosomes of patients, and significant expression of PKD1 or PKD2 between patients and controls was not observed.

The limitation in this study was the lack of the biopsy analysis of sister, which is of interest to better decipher the changes in pathologic characteristics of ARPKD. Though it had enriched lysosomal protein associated with renal lesions, the exosome proteomic analysis was based only on the proband and his sister. More cases should be enrolled in the future to identify biomarkers in exosomes associated with ARPKD.

## Conclusions

We identified parental mosaicism of *PKHD1* in a family with ARPKD. The significant pathologic characteristics were cystic dilation of proximal tubules instead of collecting ducts. In *PKHD1*-mutant urinary exosomes, expression of proteins in mitochondrial OXPHOS was downregulated markedly, whereas expression of the proteins associated with lysosomal degradation was increased sharply, including that of SGSH. These findings provide new insights in the pathophysiology underlying the polycystic kidney due to *PKHD1* deficiency.

## Data Availability Statement

The original contributions presented in the study are publicly available. This data can be found here: https://www.ncbi.nlm.nih.gov/Traces/study/?acc=PRJNA756543&o=acc_s%3Aa, http://proteomecentral.proteomexchange.org/cgi/GetDataset?ID=PXD028042 and https://www.iprox.cn/page/project.html?id=IPX0003395000.

## Ethics Statement

The studies involving human participants were reviewed and approved by the Institutional Research Ethics Committees of the Children's Hospital, Zhejiang University School of Medicine. Written informed consent to participate in this study was provided by the participants' legal guardian/next of kin.

## Author Contributions

PJ, EL, and JM conceived, supervised the project, designed the experiments, and interpreted the data. CX, QY, ZL, HS, JW, and JM carried out the clinical evaluation and patients' recruitment. CX, CY, QY, YZ, and JX performed lab investigation and data analysis. LT, ZL, and JW conducted bio-information analysis. CX and CY prepared the manuscript. PJ and JM made the final version of the manuscript. All the authors have read and approved the manuscript.

## Funding

This study was funded by the National Natural Science Foundation of China (U20A20351, 81870314) and The Major Scientific and Technological Project of Zhejiang Province (2021C03079, 2019C03028).

## Conflict of Interest

The authors declare that the research was conducted in the absence of any commercial or financial relationships that could be construed as a potential conflict of interest.

## Publisher's Note

All claims expressed in this article are solely those of the authors and do not necessarily represent those of their affiliated organizations, or those of the publisher, the editors and the reviewers. Any product that may be evaluated in this article, or claim that may be made by its manufacturer, is not guaranteed or endorsed by the publisher.
