# Supplementary material for: Mosaic PKHD1 in Polycystic Kidneys Caused Aberrant Protein Expression in the Mitochondria and Lysosomes
Source: Front Med (Lausanne). 2021 Dec 16;8:743150. doi: 10.3389/fmed.2021.743150 (PMC8716551; doi:10.3389/fmed.2021.743150)
Supplement: Supplementary file 1 [file Data_Sheet_1.PDF]

Supplementary materials: 4 Tables

Supplementary Table 1. Summary of clinical data for family members with ARPKD

Patient	Gender	Age of test (y)	Presentation	Ultrasound image	Urine routine	Scr (15-77μM)	Hypertension	CHF
Ⅱ-1	M	6	Recurrent periumbilical pain	bilateral hyperechoic renal cortex.	hematuria (+~++) proteinuria (+~++)	44	yes	no
Ⅱ-2	F	11	Abnormal urine test	bilateral hyperechoic renal cortex; multiple renal cysts	hematuria (+~+++) proteinuria (-)	50	no	no

Scr, serum creatinine; CHF, congenital hepatic fibrosis

Supplementary Table 2. Primers for Sanger sequencing

Primers	Sequences (5'---3')
PKHD1-Ex45-F	5'-AAGGGCAAGTCAATCCCATTTAAG-3'
PKHD1-Ex45-R	5'-GAGGGTCACAGATTCCATCTACC-3'
PKHD1-Ex50-F:	5'-TAGGTGGAATGATGGGGTTCCTTA-3'
PKHD1-Ex50-R	5'-TCACAGCACAAATGTCTGGAATTG-3'

Supplementary Table 3. *PKHD1* variants identified in the ARPKD family

Position[a]	Exon	Nucleotide change	Amino acid change	ACMG	Mutant type	Variant present
51750675	45	c.7205 G>A	p. G2402D	VUS	Het	I-1, II-1, II-2
51712707	50	c.7973 T>A	p. L2658*	Pathogenic	Het	I-2, II-1, II-2, II-3, II-4

[a] Genomic position is presented according to the NCBI human reference genome (hg19)

Het, heterozygous; VUS, Uncertain significance; ACMG, American College of Medical Genetics

Table S4. Antibodies in the present study

Antibodies	Source	Identifier
FPC (rabbit)	Abcam	Cat# ab122160
PC1(mouse)	Abcam	Cat# ab74115
E Cadherin (rabbit)	Abcam	Cat# ab40772
ZO1 (rabbit)	Abcam	Cat# ab221547
Acetyl-α-Tubulin (rabbit)	Cell Signaling Technology	Cat# 5335
NAGLU (rabbit)	Abcam	Cat# ab214671
SGSH (rabbit)	Proteintech	Cat# 14184-1- AP
Goat anti-rabbit IgG H+L (Alexa Fluor 488)	Beyotime	Cat# A0423
Goat anti-rabbit IgG H+L (Alexa Fluor 555)	Beyotime	Cat# A0453
